# Regulation of dendritic cell immune function and maturation by the recombinant antigen p53 of *Trichinella spiralis*

**DOI:** 10.1186/s13071-025-07074-6

**Published:** 2025-10-22

**Authors:** Xuhong Yuan, Xuejiao Yang, Caixia Han

**Affiliations:** College of Veterinary Medicine, Northeast Agricultural University, Harbin, China

**Keywords:** *Trichinella spiralis*, Dendritic cells, Indoleamine 2,3-dioxygenase, Immune response

## Abstract

**Background:**

The excretory–secretory (ES) antigen of *Trichinella spiralis* (*T. spiralis*) has garnered significant attention owing to its notable immunomodulatory activities, particularly its ability to regulate the maturation of dendritic cells (DCs). To elucidate the role of key ES components, this study employed the recombinant antigen p53 of *T. spiralis* (rTs p53) to evaluate the specific regulatory effects of this protein on DCs maturation and immune function.

**Methods:**

To dissect the immunomodulatory effect of rTs p53 on DCs, we determined its activity on DCs and its impact on indoleamine 2,3-dioxygenase (IDO) expression. Moreover, we quantitatively detected the mRNA levels and secretion levels of interleukin-6 (IL-6), interleukin-10 (IL-10), and tumor necrosis factor-alpha (TNF-α). The concentration of tryptophan in the cell supernatant was measured. In addition, we analyzed the expression levels of the DC surface molecules MHC-II, CD80, and CD86 on DCs and observed the morphology of the DCs.

**Results:**

Compared with the blank group, the ES antigen group and the rTs p53 group presented significantly increased IDO expression, a marked decrease in tryptophan concentration, and significantly upregulated mRNA transcription levels and secretion levels of IL-10, IL-6, and TNF-α. Morphologically, the DCs in the ES antigen group and the rTs p53 group exhibited more surface wrinkles but fewer spiny protrusions compared with those in the lipopolysaccharide (LPS) group, suggesting that both antigens can inhibit the maturation of DCs. In terms of surface molecules, the LPS group presented higher levels of MHC-II, CD80, and CD86 than the blank group. In the rTs p53 group, MHC-II expression was comparable to that in the blank group, and the levels of CD80 and CD86 were increased. However, the extent of this increase was significantly weaker than that in the LPS group, indicating that the rTs p53 inhibits the complete maturation of DCs.

**Conclusions:**

The findings of this study contribute to elucidating the pathogenic mechanism of *T. spiralis*, increase our understanding of its immune response, and provide a basis for exploring the underlying immune regulatory mechanisms involved.

**Graphical Abstract:**

**Figure:**
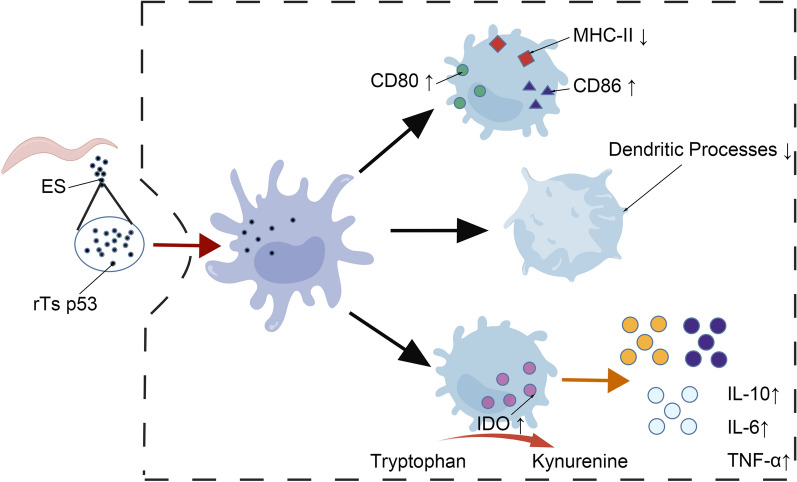

**Supplementary Information:**

The online version contains supplementary material available at 10.1186/s13071-025-07074-6.

## Background

Trichinellosis is a common zoonotic disease that seriously threatens public health and food safety [1]. Its parasitic hosts are widespread and can invade more than 150 kinds of animals, including humans [2]. Therefore, the pathogenesis and immune escape mechanism of *T. spiralis* need to be investigated.

*Trichinella spiralis* has many kinds of antigens, and their components are very complex. *Trichinella spiralis* can be used as an antigen at different developmental stages, and these antigens elicit immune responses in the host in the early stage. The excretory–secretion antigen of *T. spiralis* is secreted by rod-shaped cells, and its composition is quite complex. Gamble [3, 4] and others used monoclonal antibodies to analyze the ES antigen of muscle larvae. The results revealed that the molecular weight units of the active components of the ES antigen were 45, 49, and 53 ku, and these three components accounted for more than half of the ES antigen. The molecular weights of these three proteins are different, the secretion sites in *T. spiralis* are also different, and cross-immune reactions can occur between them [5]. The ES antigen is highly immunogenic and reactive, and it plays a key role in immunity and cyst formation. It is the most studied antigen. The rTs p53 is an important component of the ES antigen, and whether it is involved in the invasion and immune response of *T. spiralis* needs to be determined [6–8].

Dendritic cells (DCs) are the most typical antigen-presenting cells (APCs) in the immune system, and they form the link between natural immunity and acquired immunity [9–12]. In most tissues, DCs are in the “immature” state and cannot stimulate T cells. After activation, DCs enter lymph nodes, the spleen, and mucosa-related lymphoid tissues. DCs are fully mature in these tissues [12]. They affect T- and B-cell functions and maintain T lymphocyte activity [13, 14] by releasing cytokines [13]. DCs play a key role in maintaining the immune response balance [11]. DCs are crucial for parasitic infection-induced immune responses. Parasitic infections affect the maturation of DCs and induce a Th1-type response, which promotes the anti-infection ability of the host; however, the normal functions of DCs are inhibited so that parasites continue to reproduce in the host and promote immune escape [15]. DCs induce antigen-specific T-cell anergy and regulatory T-cell (Treg) production through various mechanisms. Therefore, how DCs induce and affect the immune response is highly important. Infection caused by *T. spiralis* can affect the immune response in DCs [16], but the mechanism by which *T. spiralis* induces DCs to be in such a state is unclear.

Indoleamine 2,3-dioxygenase, expressed on the surface of DCs, is a key molecule that regulates immunity [17]. IDO is the only rate-limiting enzyme in mammalian extrahepatic tissues that catalyzes the catabolism of tryptophan (Trp) via the kynurenine pathway. Trp is an essential amino acid for T lymphocyte proliferation [18]. When the expression of IDO is upregulated, the Trp metabolic pathway in the body is disrupted to different degrees, resulting in the depletion of Try, which in turn affects the function and phenotype of DCs and inhibits the activation and proliferation of T cells, resulting in T-cell anergy and immune tolerance. The overexpression of IDO suppresses T-cell growth or promotes T-apoptosis in patients with breast cancer and gastric cancer, suggesting that IDO is involved in immune tolerance and initiates immune escape [19]. In thrombocytopenia, the upregulation of IDO expression on the surface of DCs is a response to infection or tissue inflammation, which inhibits the growth and activation of T cells and induces Tregs to maintain immune tolerance [20]. In many autoimmune disorders, including autoimmune diabetes, multiple sclerosis, and rheumatoid arthritis, IDO on the surface of DCs can induce or maintain peripheral immune tolerance and immunosuppression [21].

The immune mechanism of *T. spiralis* involves the ES antigen. After being stimulated by antigens, DCs are induced to express IDO, which inhibits T-cell proliferation and then leads to immune evasion of pathogens. ES antigen can stimulate DCs to express high levels of IDO [22], indicating that IDO is closely related to the immune evasion mechanism of *T. spiralis*. During parasitic infection, DCs play an important role in preventing and eliminating invasive pathogens. The activation and maturation of DCs involve the expression of many molecules that are critical for the antigen presentation process in DCs [23]. The cytokines produced by DCs are the main components of the body that resist pathogens and play an immune role [24]. *Trichinella spiralis* parasitizes the host and regulates its immune response. DCs stimulated by the ES antigen [24] increase the secretion of antiinflammatory cytokines so that *T. spiralis* evades host immunity.

DCs maintain tolerance to autoantigens by inducing Tregs to express IDO. Intracellular IDO plays a key role in Trp metabolism, which degrades Trp into L-kynurenine, inhibits T-cell activity, and inhibits T-cell proliferation so that the antigen evades immune surveillance [25]. The metabolic activity of IDO exerts a strong effect on immunomodulation, which requires the production of biological effects by metabolizing Trp. Several studies have confirmed the role of IDO in immune regulation and the induction of tolerance [26, 27]. For example, the activity of IDO increases after malaria infection in mice, causing immunosuppression, indicating that IDO on the surface of DCs induces immune tolerance in malaria [28]. *Leishmania* infection stimulates the expression of IDO in local lymph nodes. IDO weakens the ability of DCs to stimulate T cells while inhibiting the local T-cell response to exogenous parasitic antigens, confirming that IDO can regulate the host response [29]. After in vitro induction of mouse bone marrow-derived dendritic cells (BMDCs) with diverse antigens of *Echinococcus granulosus*, the expression level of IDO increased, indicating that *E. granulosus* can evade host immune attack by regulating the level of expression of IDO on the surface of host DCs [30].

The ES antigen has high immunogenicity and reactivity and plays a key role in immunity and cyst formation. It is also the most studied antigen of *T. spiralis*. The rTs p53 is an important component of the ES antigen. Whether it is involved in the invasion and immune response of *T. spiralis* needs to be determined.

## Methods

### Recombinant proteins and cell lines

The recombinant antigen p53 of *T. spiralis* (rTs p53) was constructed and preserved by our teaching and research department. Mouse DC 2.4 cells were obtained from the cell bank of the Chinese Academy of Sciences.

### Culture of DCs

After the DC2.4 cells were thawed, they were seeded in complete RPMI-1640 medium supplemented with 10% fetal bovine serum (HyClone, Logan, Utah, USA), 100 U/mL penicillin, and 100 mg/mL streptomycin (Biological Industries) and cultured in a cell incubator at 37 °C with 5% CO_2_.

### CCK8 assay to determine the activity of DCs

DCs (1 × 10^4^ cells/well) were seeded in 96-well plates and cultured at 37 °C and 5% CO_2_ for 12 h. The experiment was started after the Trypan blue exclusion assay confirmed > 90% cell viability. The cells were then exposed to rTs p53 (5, 10, 15, 20, 30, or 45 µg/mL) or an equal volume of phosphate buffered saline (PBS) for 24 h. Each condition was run in triplicate; the corresponding cell-free wells served as background controls. After stimulation, the medium was replaced with 100 µL of fresh medium containing 10% Cell Counting Kit-8 (CCK-8) reagent (Thermo Fisher Scientific), and the mixture was incubated for 1 h at 37 °C. The absorbance at 450 nm was measured with a microplate reader; viability was calculated after background values were subtracted and expressed as a percentage of the PBS control.

### Effects of different concentrations of the recombinant antigen p53 of *T. spiralis* on IDO expression in DCs

The rTs p53 was used to stimulate the cells for 24 h according to the concentration gradient (0, 5, 10, 15, 20, 30, and 45 μg/mL), and 1000 U/mL mouse interferon-gamma (IFN-γ) was used to stimulate the cells as a positive control, with a blank control group. After stimulation, total cellular RNA was separated via a one-step method using TRIzol reagent (Takara Bio Co., Ltd., Beijing, China), and the RNA content and quality were analyzed using a Nano-300 micro-spectrophotometer (Allsheng, Hangzhou, China). The reverse-transcribed cDNA was detected via fluorescence quantitative qPCR. Statistical analysis was performed using the 2^−ΔΔCt^ method.

### Time effect of the recombinant antigen p53 of *T. spiralis* on the expression of IDO in DCs

DCs (1 × 10^6^/mL) were seeded in six-well plates. These DCs were stimulated with 20 µg/mL rTs p53, 1000U/mL mouse IFN-γ-stimulated cells were used as a positive control, and a blank control group was set up. After 0, 3, 6, 12, 24, and 36 h of stimulation, the cells from each group were harvested and collected for total protein extraction using a protein isolation kit (KeyGEN BioTECH Co., Ltd., Nanjing, China). The extracted proteins were separated via 12% sodium dodecyl sulfate–polyacrylamide gel electrophoresis (SDS-PAGE) and subsequently transferred onto polyvinylidene fluoride (PVDF) membranes. To block nonspecific binding, the membranes were incubated with 5% (w/v) nonfat milk powder dissolved in tris-buffered saline with tween 20 (TBST) buffer (0.02% Tween 20, 150 mmol/L NaCl, and 20 mmol/L Tris–HCl, pH 7.6). After the samples were sealed, the membranes were incubated with a primary mouse IDO monoclonal antibody (1:2000 dilution) and a mouse beta-actin monoclonal antibody (1:8000 dilution, reference control) (Peprotech, Rocky Hill, NJ, USA) at 4 °C overnight.

The primary antibody was recovered the following day, and the samples were washed with TBST three times for 15 min each. After the samples were washed, the secondary antibody (goat anti-rabbit IgG (HRP)) was added to the closed solution at a dilution ratio of 1:4000, and the samples were incubated for 2 h on a shaking table at ambient temperature. After an additional set of TBST washes, enhanced chemiluminescence (ECL) detection (HaiGene Co., Ltd., Harbin, China) was performed according to the manufacturer’s instructions. IDO expression was quantified using ImageJ software, with beta-actin serving as an internal control for normalization.

### Assessment of IDO expression through immunofluorescence

The DCs (5 × 10^5^/well) were spread on a 12-well plate. The cells were stimulated with ES antigen, rTs p53, or mouse IFN-γ, and a blank control group was used. After 24 h, each well was washed with PBS three times. Thereafter, the cells were fixed with 4% paraformaldehyde. After 15 min at ambient temperature, 400 µL of 0.2% Triton X-100 was added to disrupt the cell membrane, which was subsequently closed for 30 min (prepared with 2% bovine serum albumin (BSA) as the cell sealing solution). After closure, the prepared primary antibody IDO (diluted in PBS at 1:300; Bioss, Beijing) was directly added and incubated overnight at 4 °C in the dark. The following day, the sections were incubated with the fluorescent secondary antibody in PBS three times. After the secondary antibodies were collected, they were washed with filtered phosphate-buffered saline with tween 20 (PBST) twice, and DAPI (2 g/mL) was added for counterstaining for 5 min. After the filtered PBST was washed three times, 500 µL of PBS was added to each well and detected by fluorescence microscopy (Agilent Technologies, Winooski, VT, USA). All the experiments were repeated three times.

### Detection of the expression levels of IL-10, IL-6, and TNF-α in DCs by RT-PCR and ELISA

The cells were inoculated in a six-well plate at a density of 1 × 10^6^cells/mL, and the cells were stimulated with ES antigen, rTs p53, or 1000 U/mL mouse IFN-γ (positive control). A blank control group was also included. After 0, 3, 6, 12, 24, and 36 h of stimulation, the cells in each group were collected to obtain total RNA from each group, which was subsequently reverse-transcribed into cDNA using a reverse transcription kit (Promega, Madison, WI, USA). The mRNA transcript levels of IDO, IL-10, IL-6, and TNF-α in cells from each group were dynamically detected using qPCR, with *β*-actin serving as the reference gene. All primers were synthesized by Kumei Biotechnology Co., Ltd., and RT-PCR was performed with a ChamoQ universal SYBR qPCR Master Mix kit (Vazyme Biotech Co., Ltd., Nanjing, China). The data obtained were examined using the 2^−ΔΔCt^ method. The culture supernatants were centrifuged to remove debris, and secreted IL-10, IL-6, and TNF-α protein levels were quantified using enzyme-linked immunosorbent assay (ELISA) (Enzyme-linked Biotechnology Co., Ltd., Shanghai, China) kits.

### Determination of tryptophan concentration via high-performance liquid chromatography

The cells (1 × 10^6^ cells/well) were plated in six-well plates. The cells were stimulated with ES antigen, rTs p53, or 1000 U/mL mouse IFN-γ (positive control group), and a blank control group was established simultaneously. After the cells were cultured for 24 h, a standard curve for Trp was generated at concentrations of 10, 20, 40, 60, 80, and 100 μM on the basis of peak areas measured by high-performance liquid chromatography (HPLC; Agilent Technologies, Santa Clara, CA, USA). Subsequently, the DCs were treated with each antigen group for various durations. The peak area of Trp in the DC supernatant was quantified via high-performance liquid chromatography (HPLC), and concentrations were calculated via a standard curve. All Trp standard working solutions were analyzed in triplicate, and the mean values were used for analysis. Correlation and regression analyses were performed using the least squares method, with the standard solution concentration as the independent variable and the peak area as the dependent variable, to derive a regression equation and construct a standard curve.

### Determining the effects of the recombinant antigen p53 of *T. spiralis* on the morphology and surface molecules of DCs

#### Detection of the expression of cell surface molecules by flow cytometry

The cells were spread on a six-well plate at a density of 1 × 10^6^ per well. The cells were stimulated with ES antigen, rTs p53, or 1000 U/mL mouse IFN-γ (positive control group); a blank control group was also established. After 24 h of culture, the culture medium was discarded, the cells were washed twice with PBS, and the cells were collected in flow cytometry tubes. Fluorescently labeled antibodies (anti-MHC-II PE, anti-CD86 APC, and anti-CD80 FITC) (Tonbo Biosciences Co., Ltd., San Diego, USA) were introduced at 0.25 g/100 μL. In contrast, PBS, instead of antibodies of identical volume, was added to the blank control group. After mixing, the mixture was incubated for 35 min in the dark at 4 °C. Following incubation, the cells were rinsed with PBS before centrifugation, and the supernatants were discarded. Finally, cell resuspension was performed with 500 μL of PBS, followed by filtration with a nylon filter membrane and detection by flow cytometry (BD Biosciences, USA). All the assays were conducted three times.

#### Observation of cell morphology by scanning electron microscopy

The DCs were seeded in six-well plates (cell concentration of 1 × 10^6^cells/mL), and the DCs were stimulated with 20 µg/mL rTs p53 and 5 µg/mL ES antigen, with LPS (200 ng/mL) used as the positive control. A blank control group was also set up, and after stimulation for 24 h, the samples were fixed, rinsed, dehydrated, replaced, dried, stuck to the sample, coated, and observed, after which the cell state was recorded. The DCs in every group were selected at random, and the DCs with protrusions were subsequently counted. In total, 10 DCs containing protrusions were randomly selected from each group, and the protrusions on the surface of each cell were counted.

### Data statistics and analysis

The experimental data were processed using SPSS 24.0 and GraphPad Prism 8.3. The data are presented as the means ± standard deviations (means ± SDss). Intergroup differences were determined by one-way and two-way analysis of variance (ANOVA). A *P*-value less than 0.05 was considered statistically significant.

## Results

### Effect of rTs p53 on the proliferation of DCs

**Fig. 1:**
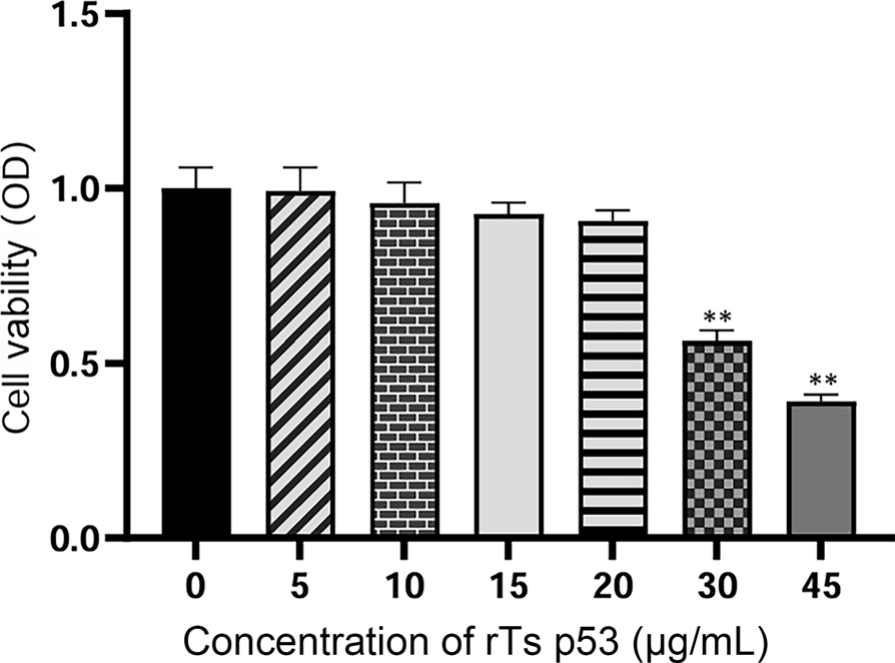
Effect of recombinant antigen p53 of *T. spiralis* (rTs p53) on dendritic cell activity. Dendritic cells (DCs) were treated with rTs p53 at concentrations ranging from 0 to 45 μg/mL for 24 h. Cell viability was assessed using the CCK-8 assay, and the optical density (OD) was measured at 450 nm. Data are presented as the mean ± SD of triplicate measurements from one representative experiment out of three independent experiments performed. **P < 0.01 versus the untreated control group (0 μg/mL)

After 24 h of rTs p53 treatment at different doses (Fig. 1), the proliferation of DCs remained unaffected at doses < 20 μg/mL, and the proliferation of DCs decreased significantly at concentrations of 30 μg/mL and 45 μg/mL compared with that of the blank control group (ANOVA, *F* (6,14) = 12.78,*P* < 0.01).

### The recombinant antigen p53 of *T. spiralis* significantly upregulates the expression of IDO in DCs

**Fig. 2:**
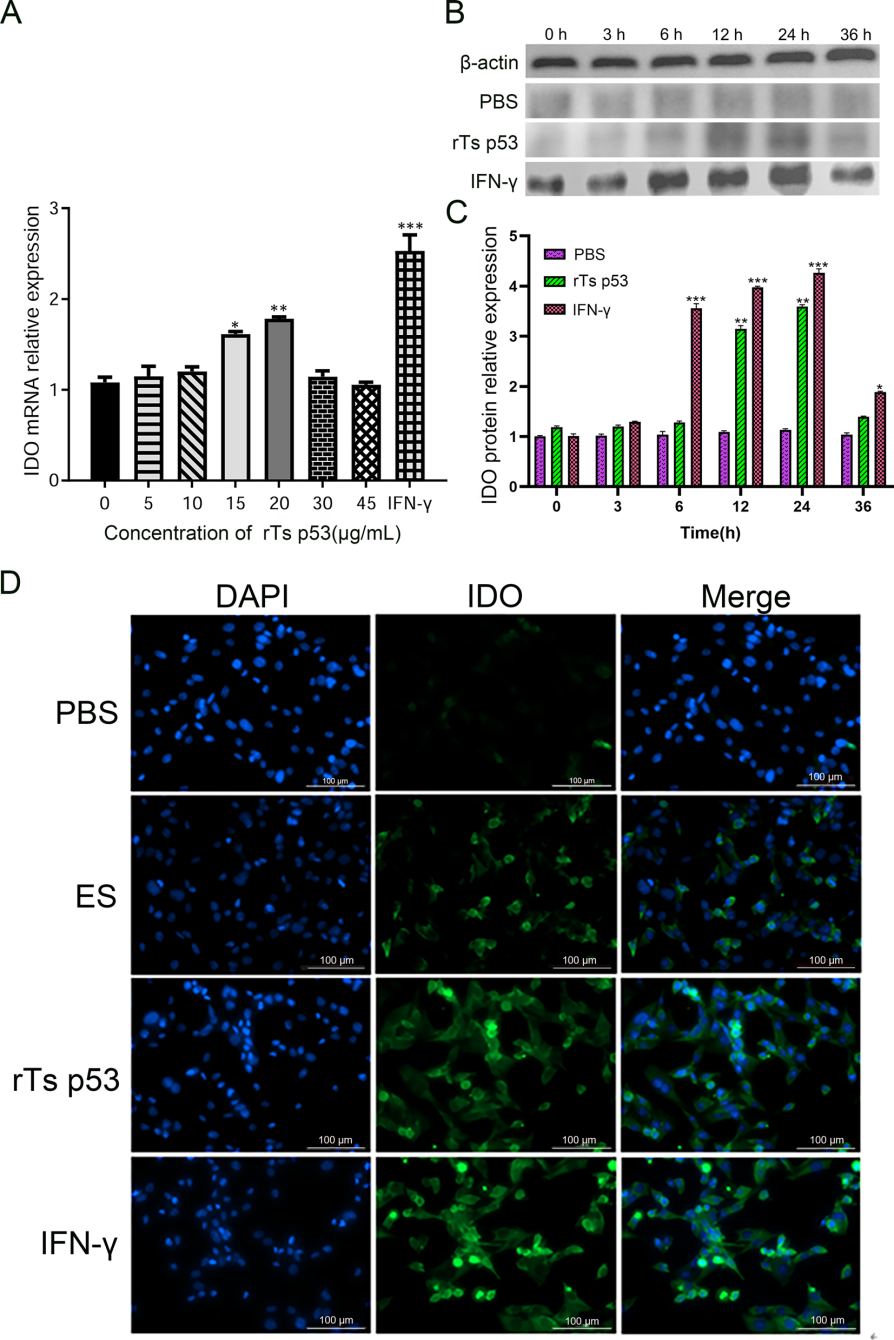
rTs p53 induces indoleamine 2,3-dioxygenase (IDO) expression in dendritic cells (DCs). **A** DCs were stimulated with 5–45 µg/mL rTs p53 for 24 h. IDO mRNA levels were quantified by quantitative real-time PCR (qRT-PCR). **B** Representative western blot images of IDO protein expression. **C** Quantitative analysis of IDO protein levels from (**B**) normalized to β-actin. **D** IDO expression was detected by indirect immunofluorescence assay. Interferon-gamma (IFN-γ) was used as a positive control. Data are presented as the mean ± SD of three independent experiments. *P < 0.05, **P < 0.01, ***P < 0.001 versus the untreated control group. Scale bar: 100 μm

To determine the optimal conditions for rTs p53-induced IDO expression in DCs, we first stimulated the cells for 24 h with increasing concentrations (5–45 μg/mL). IDO mRNA remained unchanged below 10 μg/mL, increased significantly at 15 μg/mL, and peaked at 20 μg/mL (ANOVA, *F* (7, 16) = 6.89, *P* < 0.01) (Fig. 2A). Therefore, 20 μg/mL was selected as the working concentration that robustly upregulates IDO while preserving DC viability. Next, we fixed the concentration at 20 μg/mL and examined the time course from 0 to 36 h (Fig. 2B, C). The IDO protein levels were unchanged during the first 6 h, increased markedly at 12 h (ANOVA, *F* (5, 12) = 4.46, *P* < 0.01), peaked at 24 h (ANOVA, *F* (5, 12) = 4.46, *P* < 0.01), and remained elevated at 36 h (ANOVA, *F* (5, 12) = 4.46, *P* < 0.05). Thus, stimulation with 20 μg/mL rTs p53 for 24 h represents the optimal condition for inducing IDO expression in DCs. Consistent with these findings, indirect immunofluorescence analysis of IDO expression in DCs stimulated with different inducers revealed that after 24 h of induction, a large amount of green fluorescence appeared in the cells of the IFN-γ-positive control group. Green fluorescence was detected in the cytoplasm of DCs treated with the ES antigen and rTs p53, whereas green fluorescence was negligible in the blank control group. These findings further confirmed that the rTs p53 can upregulate IDO in DCs (Fig. 2D).

### The recombinant antigen p53 of *T. spiralis* significantly reduces the tryptophan concentration in the cell culture supernatant

As shown in Table 1, when DCs were stimulated with rTs p53 for 3 h, the concentration of Trp (24.33 ± 1.09 μmol/L) remained unchanged relative to that in the blank control group (25.5 ± 0.15 μmol/L). When DCs were stimulated with rTs p53 for 6 h, the concentration of Trp (23.01 ± 0.87 μmol/L) did not significantly differ from that in the blank control group (23.3 ± 0.98 μmol/L). However, the Trp concentration (19.46 ± 0.25 μmol/L) in the IFN-γ treatment group was not significantly different from that in the blank control group (23.3 ± 0.98 μmol/L) (ANOVA, *F* (3,6) = 15.67, *P* < 0.05). After intervention for 12 h, the Trp concentration (18.13 ± 0.23 μmol/L) in the ES antigen treatment group was lower than that in the blank control group (20.58 ± 0.45 μmol/L), with no significant difference (ANOVA, *F* (3,6) = 25.34, *P* < 0.05); moreover, the Trp concentration (12.99 ± 1.71 μmol/L) in the rTs p53 treatment group was significantly lower than that in the blank control group (ANOVA, *F* (3,6) = 32.87, *P* < 0.01). The Trp concentration (11.52 ± 0.30 μmol/L) in the IFN-γ treatment group was significantly different from that in the blank control group (ANOVA, *F* (3,6) = 29.54,* P* < 0.01). Following intervention for 24 h, relative to those in the blank control group (19.16 ± 0.51 μmol/L), the concentrations of Trp in the ES antigen treatment group (14.38 ± 0.28 μmol/L), the rTs p53 treatment group (9.41 ± 0.28 μmol/L), and the IFN-γ treatment group (7.17 ± 0.18 μmol/L) decreased significantly (ANOVA, *F* (3,6) = 38.92, *P* < 0.01). After 36 h of intervention, the level of the rTs p53 decreased significantly relative to that in the blank control group. The Trp concentration in the ES antigen treatment group (9.37 ± 0.76 μmol/L), the rTs p53 treatment group (8.41 ± 0.23 μmol/L), and the IFN-γ treatment group (6.26 ± 0.47 μmol/L) decreased significantly (ANOVA, *F* (3,6) = 74.815, *P* < 0.001)*.*

**Table 1:** Tryptophan concentration of different treatment groups

h	Tryptophan concentration μmol·L^−1^			
	Control group	ES group	rTs p53 group	Positive group
0 h	26.66 ± 0.77	25.43 ± 0.84	25.37 ± 0.59	24.5 ± 1.04
3 h	25.5 ± 0.15	24.98 ± 0.47	24.33 ± 11.09	23.78 ± 0.18
6 h	23.3 ± 0.98	21.04 ± 0.59	23.01 ± 0.87	19.46 ± 0.25*
12 h	20.58 ± 0.45	18.13 ± 0.23*	12.99 ± 1.71**	11.52 ± 0.30**
24 h	19.16 ± 0.51	14.38 ± 0.28*	9.41 ± 0.28**	7.17 ± 0.18**
36 h	17.43 ± 0.85	9.37 ± 0.76**	8.61 ± 0.23**	6.26 ± 0.47**

Compared with control group, *P < 0.05, **P < 0.01

### The recombinant antigen p53 of *T. spiralis* enhances the expression of DCs cytokines in a time-dependent manner, and the effect is affected by IDO

**Fig. 3:**
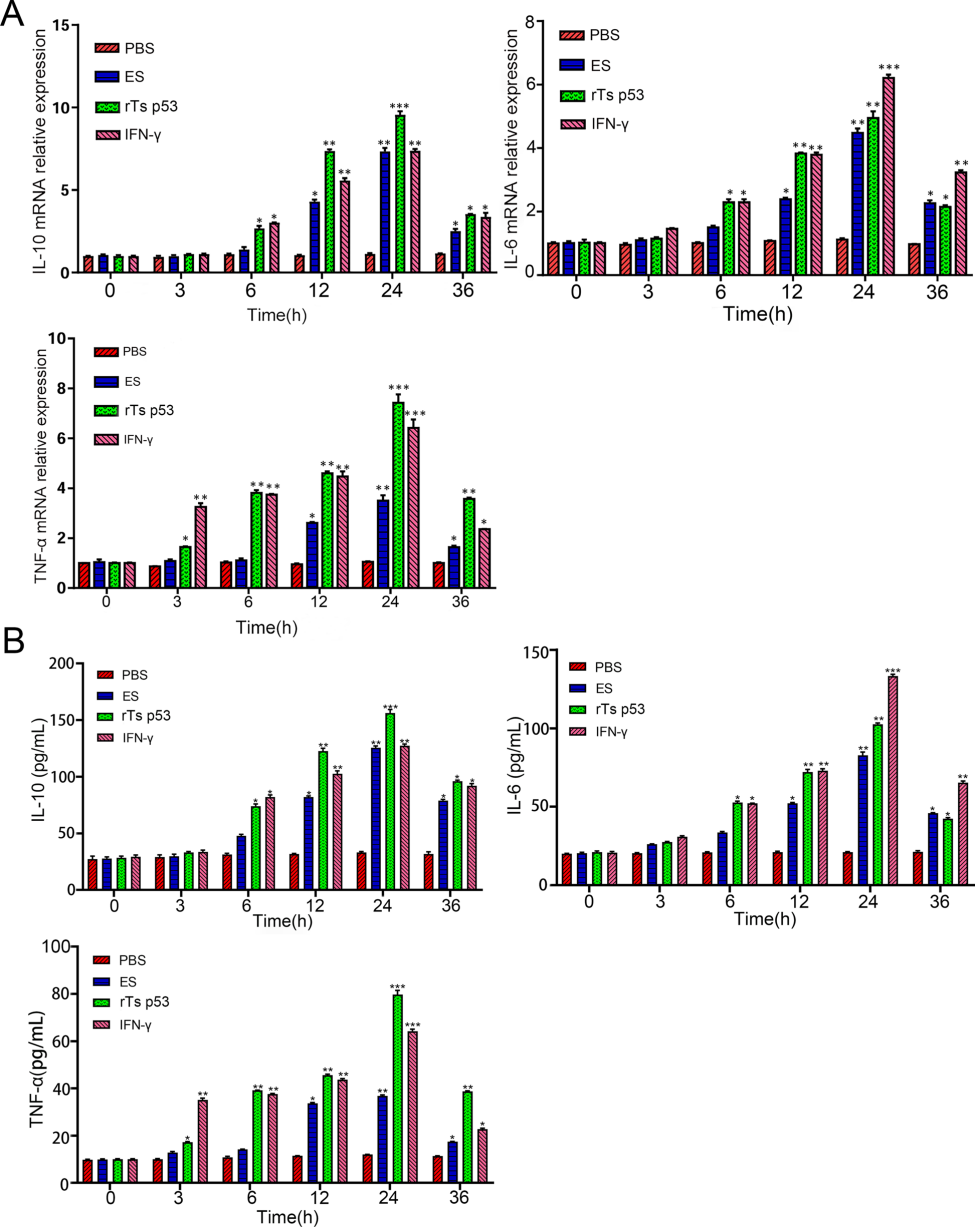
rTs p53 modulates cytokine production in dendritic cells (DCs). **A** DCs were stimulated with rTs p53 (20 µg/mL). The relative mRNA expression levels of interleukin-10 (IL-10), interleukin-6 (IL-6), and tumor necrosis factor-alpha (TNF-α) were determined by qRT-PCR at 6, 12, and 24 h poststimulation. **B** The concentrations of IL-10, IL-6, and TNF-α in the culture supernatants were measured by enzyme-linked immunosorbent assay (ELISA) at the same time points. Data are presented as the mean ± SD of three independent experiments. Interferon-gamma (IFN-γ) was used as a positive control. *P < 0.05, **P < 0.01, ***P < 0.001 versus the unstimulated control group at the respective time point

To determine how rTs p53 affects the levels of IL-10, IL-6, and TNF-α in DCs, we used qPCR and ELISA to measure the cytokine levels in DCs after rTs p53 stimulation (Fig. 3A, B). Following stimulation with 20 μg/mL rTs p53, the mRNA and protein levels of IL-6 and IL-10 did not significantly change within 0–3 h. Their levels increased continuously from 6 to 24 h and peaked at 24 h, at which point the increases were statistically significant (IL-6: ANOVA,*F* (3, 12) = 15.87, *P* < 0.001; IL-10: ANOVA, *F* (3, 12) = 9.45, *P* < 0.01). Although they decreased at 36 h, the levels remained higher than those in the control group (IL-6: ANOVA, *F* (3, 12) = 5.12, *P* < 0.05; IL-10: ANOVA, *F* (3, 12) = 4.89, *P* < 0.05). The expression of TNF-α significantly increased as early as 3 h after stimulation (ANOVA, *F* (3, 12) = 6.78, *P* < 0.01). TNF-α expression peaked at 24 h (ANOVA, *F* (3, 12) = 35.20, *P* < 0.001) and remained high at 36 h (ANOVA, *F* (3, 12) = 28.15, *P* < 0.001). The ES antigen showed a similar but weaker trend. The positive control IFN-γ significantly induced TNF-α expression as early as 3 h. To statistically confirm the overall effect of rTs p53 treatment across all time points, two-way ANOVA was performed. The analysis revealed a significant interaction effect between treatment and time for all cytokines (IL-6: ANOVA, *F* (15, 80) = 4.05, *P* < 0.001; IL-10: ANOVA, *F* (15, 80) = 3.88, *P* < 0.001; TNF-α: ANOVA, *F* (15, 80) = 18.32, *P* < 0.001), indicating that the effect of rTs p53 on cytokine expression depended on the duration of stimulation. In summary, rTs p53 significantly promoted the expression of DC cytokines, suggesting that it is involved in immune regulation by regulating cytokines.

**Fig. 4:**
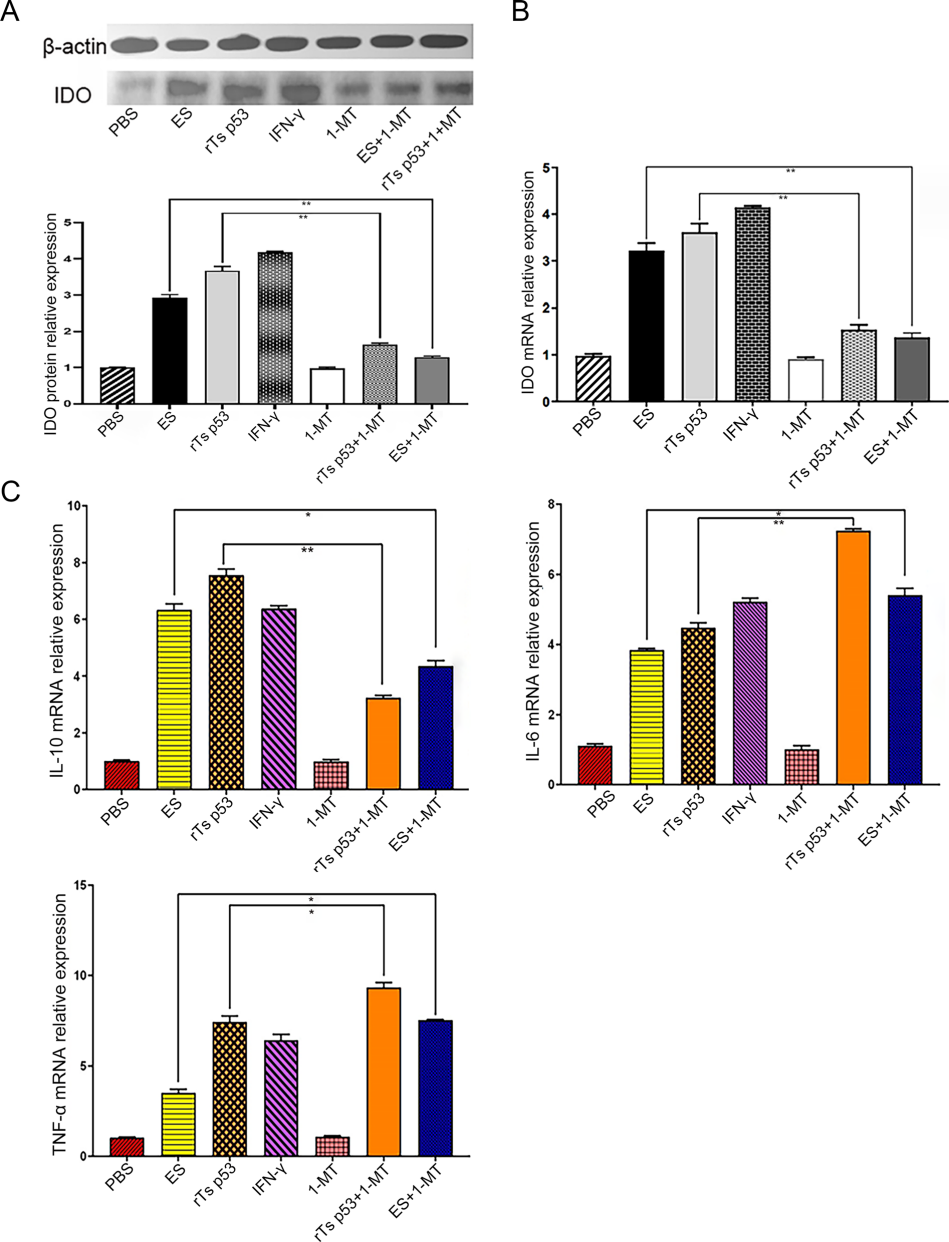
The level of expression of each factor after 1-MT inhibition. **A** Western blot analysis of the expression of IDO in different groups and relative gray values; **B** Real-time RT-PCR was performed to identify the relative expression of IDO. **C** Real-time RT-PCR was performed to identify the relative expression of interleukin (IL)−6, IL-10, and tumor necrosis factor (TNF)-α mRNAs in DCs after 1-MT inhibition. IFN-γ was used as a positive control in these experiments. *P < 0.05, **P < 0.01

Further validation was subsequently conducted to explore the regulatory role of IDO in this process (Fig. 4A). Following 1-MT pretreatment, DCs stimulated with ES antigen and rTs p53 exhibited significantly reduced IDO expression at both protein and transcriptional levels compared with antigens alone without 1-MT treatment (ANOVA, *F* (6, 14) = 15.72, *P* < 0.01). In DCs pretreated with 1-MT followed by stimulation with ES antigen, the transcriptional level of IL-10 mRNA was significantly reduced compared with DCs stimulated with ES antigen alone, and was even lower than that in unstimulated control cells (ANOVA, *F* (4, 8) = 7.01, *P* < 0.01) (Fig. 4B). Concurrently, the mRNA levels of IL-6 and TNF-α showed a marked increase (IL-6: ANOVA, *F* (4, 8) = 7.46, *P* < 0.01; TNF-α: ANOVA, *F* (4, 8) = 4.46, *P* < 0.05). These results indicate that IDO is involved in the stimulation of DCs by the rTs p53 and leads to the expression of inflammatory factors.

### The recombinant antigen p53 of *T. spiralis* induces incomplete maturation of DCs

**Fig. 5:**
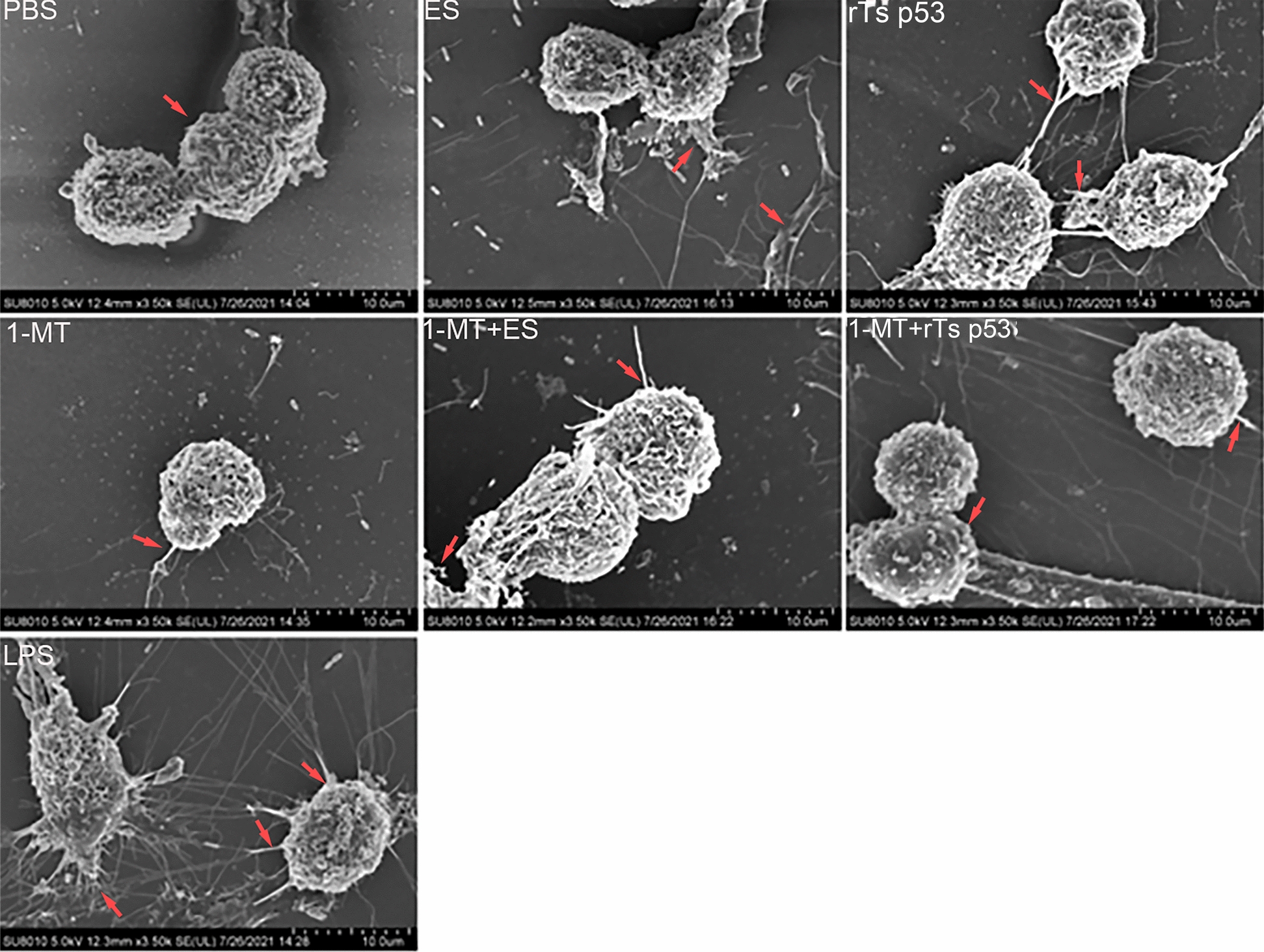
The morphological features of DCs were evaluated after stimulation with ES antigen, rTs p53, 1-MT, a combination of ES and 1-MT, a combination of rTs p53 and 1-MT, or LPS. Cellular morphology was examined using scanning electron microscopy. LPS and PBS were employed as the positive and blank controls, respectively

To intuitively determine how the rTs p53 affects DC maturation, the morphology of cells stimulated with the rTs p53 was examined by scanning electron microscopy (Fig. 5). The results revealed that there were almost no burr-like processes on the surface of the DCs in the blank group, which were immature DCs. In the positive control LPS group, many burr-like processes were observed on the surface of the DCs, which were clear and slender, indicating that these were mature DCs. Compared with that in the blank control group (the PBS group), the number of cell surface wrinkles in the rTs p53 group was greater. However, the number of surface burr-like processes was considerably lower than that in the LPS group, which indicated that rTs p53 stimulated DCs and that the maturation of DCs was inhibited.

Subsequently, 100 cells were randomly selected from each group, the number of cells with protrusions on the surface was counted, and parallel experiments were conducted three times. As shown in Table 2, in the ES antigen group (24 protuberant cells), rTs p53 group (22 protuberant cells), and blank control group (22 protuberant cells), the proportions of mature DCs in the ES antigen and rTs p53 groups were low. In contrast, a high proportion of mature DCs was found in the LPS group (ANOVA, *F* (6, 14) = 852.4, *P* < 0.01). Then, 10 DCs with protrusions from each group were randomly selected to count the number of protrusions on their surfaces. The results are shown in Table 3. The average number of protrusions in DCs in the rTs p53 group increased relative to that in the blank control group. However, the number of protrusions still decreased significantly compared with that in the LPS group (ANOVA, *F* (6, 63) = 125.6, *P* < 0.01). These findings indicate that the rTs p53 cannot effectively stimulate the maturation of DCs. We then further investigated the possibility that IDO is involved in regulating this process (Fig. 5). The rTs p53 + 1-MT group presented results similar to those of the rTs p53 group. DCs displayed increased surface folds, but the number of spiny protrusions remained lower in the rTs p53 + 1-MT group than in the LPS group, indicating an immature phenotype. Pretreatment with 1-MT (rTs p53 + 1-MT group) did not alter the effect of rTs p53; the number of protrusions was comparable between the two groups (Table 2), as both groups presented an immature morphology with fewer protrusions. Statistical analysis of surface protrusions from 10 representative DCs per group (Table 3) revealed no significant difference between the rTs p53 and rTs p53 + 1-MT groups, confirming that rTs p53 induces incomplete DC maturation via an IDO-independent mechanism.

**Table 2:** Comparison of the number of dendrite cells (DCs) with dendrites in different groups (random selection 100 DCs in each group)

Group	The number of DCs	The number of DC with dendrites
Control	100	21.8 ± 0.84
ES	100	23.7 ± 1.8*ͻͻ
rTs p53	100	25.8 ± 1.67*ͻͻ
LPS	100	88.43 ± 4.9
1-MT	100	20.33 ± 1.48
1-MT ± ES	100	23.45 ± 3.52*ͻͻ
1-MT ± rTs p53	100	26.24 ± 1.57*ͻͻ

Compared with control group, *P < 0.05

Compared with LPS group, **P < 0.01

**Table 3:** DC with dendrites of different groups comparative with each other (selection 10 DCs with dendrites in each group)

Group	The number of DCs	The number of dendrites
Control	10	5.23 ± 0.64
ES	10	7.23 ± 0.48*ͻͻ
rTs p53	10	6.47 ± 4.33*ͻͻ
LPS	10	37.45 ± 2.45
1-MT	10	5.84 ± 1.46
1-MT ± ES	10	6.31 ± 0.68*ͻͻ
1-MT ± rTs p53	10	7.43 ± 2.75*ͻͻ

Compared with control group, *P < 0.05

Compared with LPS group, **P < 0.01

### The recombinant antigen p53 of *T. spiralis* blocks dendritic cell maturation by downregulating MHC-II expression

**Fig. 6:**
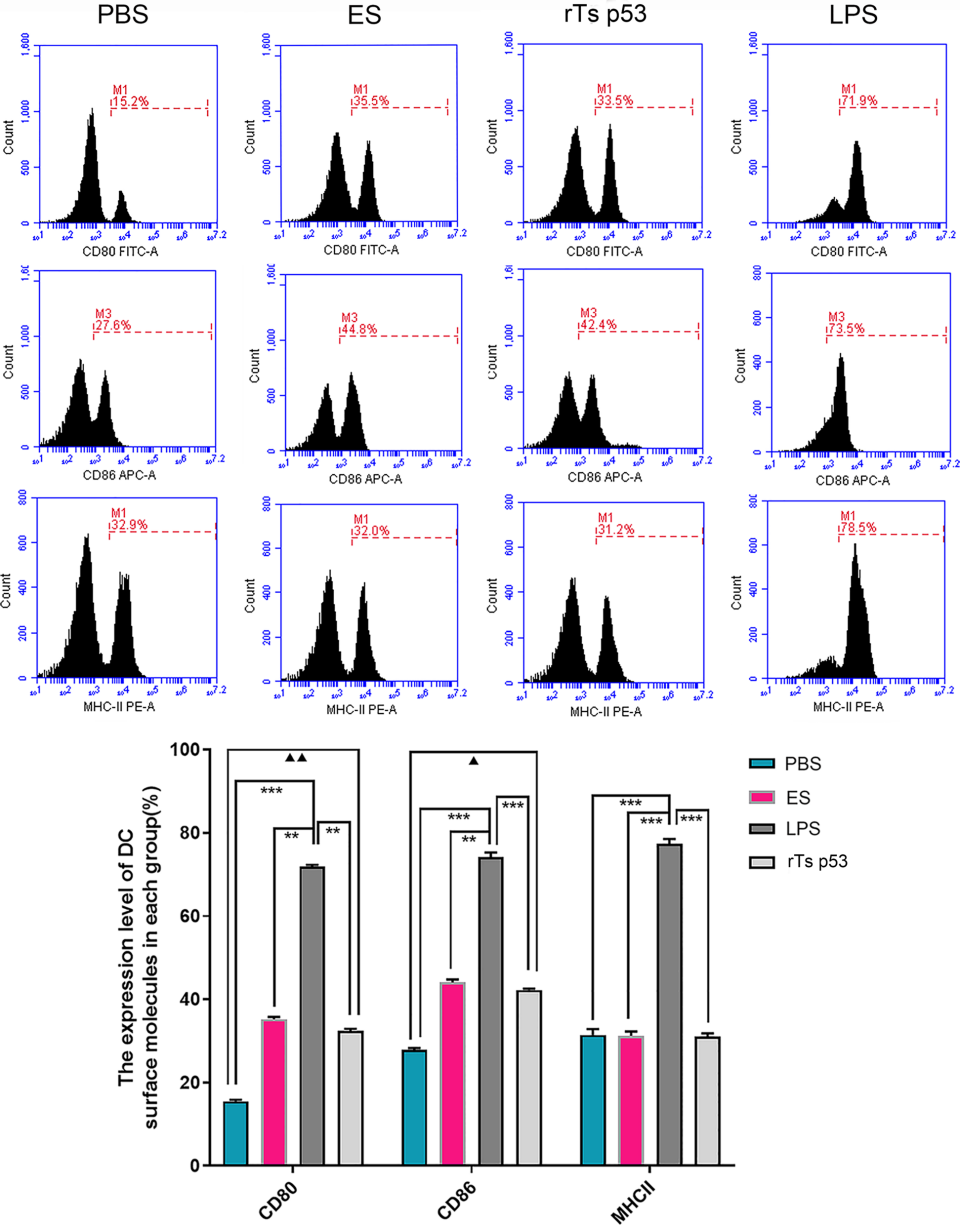
Expression of DC surface molecules in different treatment group. DCs was stimulated by ES antigen, rTs p53 and LPS. The expressions of MHC-II, CD80 and CD86 in each group were detected by flow cytometry, and the results were analyzed quantitatively. Compared with the control group, *▲P* < 0.05; *▲▲P* < 0.01; Compared with LPS group, **P < 0.01; **P < 0.001

MHC-II molecules, the B7 family (CD80, CD86), and other molecules are the key indicators of the maturity of DCs. The expression of molecules on the surface of immature DCs is very low, and once activated by external antigens, the expression of molecules on the surface of cells increases. The DCs were stimulated with different antigens, MHC-II, CD80, and CD86 were detected by flow cytometry, and the results were quantified (Fig. 6). In the blank control group, 32.9% of the cells expressed MHC-II, 15.2% expressed CD80, and 27.6% expressed CD86, indicating that the DCs exhibited an immature phenotype. However, after LPS stimulation, the DCs were fully mature, and the percentages of cells containing the surface molecules MHC-II, CD86, and CD80 were 78.9%, 73.5%, and 71.9%, respectively. Compared with that in the blank control group, the surface expression of MHC-II in DCs was not significantly different (ANOVA, *F* (3, 16) = 28.75,* P* > 0.05), whereas CD86 expression was increased (ANOVA, *F* (3, 16) = 35.2, *P* < 0.05), and CD80 expression was significantly upregulated (ANOVA, *F* (3, 16) = 45.6, *P* < 0.01). However, compared with that in the LPS group, this increase was inhibited, and the difference was significant (MHC-II: ANOVA, *F* (3, 16) = 28.75,* P* < 0.001; CD86: ANOVA, *F* (3, 16) = 35.2, *P* < 0.001; CD80: ANOVA, *F* (3, 16) = 45.6, *P* < 0.001). The results revealed that the rTs p53 inhibited the expression of MHC-II on DCs, affected the complete maturity of DCs, and hindered their immune function. This finding was consistent with the results observed via electron microscopy.

## Discussion

Trichinellosis is caused by *T. spiralis*, which affects not only human health but also animal production and food safety. The focus of research on the *T. spiralis* immune mechanism is ES antigens, and the rTs p53, an important component of ES antigens, plays a crucial role in assessing the pathogenic mechanism of *T. spiralis*.

DCs are pivotal regulators of the host immune response to *T. spiralis* [31]. Upon exposure to ES antigens, DCs upregulate IDO. Studies have shown that IDO is the rate-limiting enzyme in Trp catabolism and plays a pivotal role in DC biology [32]. In the present study, maximal upregulation of IDO in DCs was observed after 24 h of stimulation with rTs p53 at 20 µg/mL. HPLC analysis of culture supernatants confirmed a significant decrease in extracellular Trp at 12 h and 24 h poststimulation, demonstrating that rTs p53-driven IDO overexpression depletes Trp. Although previous reports have linked Trp deprivation to T-cell proliferation arrest, Treg induction, or Th1 apoptosis [33, 34], these downstream effects were not directly examined here.

DC-secreted cytokines are pivotal determinants of both the quality and magnitude of antiparasitic immunity [35]. Persistent stimulation of DCs by ES antigens may induce immune tolerance through IDO-mediated suppression of proinflammatory cytokines [27]. In our experiments, rTs p53 skewed DCs toward an antiinflammatory cytokine profile, whereas blockade of IDO with 1-MT reversed this bias and promoted proinflammatory cytokine release. These data indicate that IDO-dependent “antiinflammatory microenvironments” favor chronic parasite survival.

Various helminth antigens induce distinct DC phenotypes, yet a common feature is partial maturation [36–38]. Most studies have shown that helminths impair DC maturation, thereby limiting antigen presentation [37]. While elucidating the IDO-mediated antiinflammatory role of rTs p53, we further investigated whether this pathway likewise influences DC maturation. Scanning electron microscopy revealed that DCs exposed to the ES antigen or rTs p53 presented sparse surface protrusions characteristic of semimature or immature cells, with no significant differences between the IDO-competent and IDO-inhibited groups. Flow cytometric analysis confirmed low MHC-II expression on ES-stimulated DCs. Our findings are consistent with those of previous studies on the functional effects of parasite-derived antigens on dendritic cell phenotypes [39–44], demonstrating that they upregulate the expression of several DC surface markers but not all. This study demonstrated that DC maturation defects are independent of IDO expression, suggesting the existence of other regulatory mechanisms. This aspect was not further investigated in the current study.

This study demonstrated that the rTs p53 plays a significant role in modulating DC immunoregulatory functions, although further *in vivo* validation is needed. Our work clearly establishes the critical role of IDO in rTs p53-mediated immunomodulation of DCs while delineating the specific scope of IDO involvement. In addition, the rTs p53 interferes with DC maturation through other regulatory mechanisms, laying the foundation for understanding the complex regulatory network of rTs p53 in DC maturation and providing potential therapeutic targets for the prevention and treatment of trichinellosis.

## Conclusions

Our study is the first to systematically demonstrate that the rTs p53 modulates DCs by inducing high expression of IDO. Furthermore, we observed that the rTs p53 inhibits the expression of MHC-II molecules on the surface of DCs and impedes their full maturation. This process is independent of the IDO pathway. These findings reveal the critical role of the rTs p53 in the immunomodulatory mechanisms of *T. spiralis*, providing a novel theoretical basis and experimental evidence for understanding parasite–host interactions. Further *in vivo* studies are needed to confirm these findings and elucidate the underlying mechanisms involve.

## Supplementary Information

Supplementary Material 1. The primers used in the qRT-PCR experiment

## Data Availability

Data supporting the main conclusions of this study are included in the manuscript.

## Abbreviations

ES antigen: Excretory–secretory antigen of *Trichinella spiralis*
rTs p53: Recombinant antigen p53 of *Trichinella spiralis*
IDO: Indoleamine 2,3-dioxygenase
MHC-II: Major histocompatibility complex class II molecule
CD80: Cluster of differentiation 80
CD86: Cluster of differentiation 86
