# Supplementary material for: Regulation of dendritic cell immune function and maturation by the recombinant antigen p53 of Trichinella spiralis
Source: Parasit Vectors. 2025 Oct 22;18:423. doi: 10.1186/s13071-025-07074-6 (PMC12542060; doi:10.1186/s13071-025-07074-6)
Supplement: Supplementary file 1 — Supplementary Material 1. The primers used in the qRT-PCR experiment [file 13071_2025_7074_MOESM1_ESM.pdf]

The primers used in the qRT PCR experiment.

mouse IDO (registration number: NM_008324), F: 5'-AGCAATCCCCACTGTATCCA-3’, R: 5'-GGTCCACACAAAGTCACGCATC-3’;

IL-10 (registration number: NM_010548.2), F: 5'-GTAGAGTTGCCCAGC-3’, R: 5'-CACCTTGGTCTGGGAGCTTAT-3’;

IL-6 (registration number: NM_001314054.1), F: CACATGTTCTCTGGGAAATCG, R: TTGTATCTCGAAGTTTCAGATTGT;

TNF-α (registration number: NM_001278601.1), F: 5'-TCTTCCTTCCTTGG, R: 5'-CATTGGTTGGTTTTGCTCCAGA,

β-actin (registration number: NM_007393.5) F: GGCTTTTTCCTTCCTCG, R: CCAGTTGGTAACAAATGCCATGT,
